# 2,9-Dichloro-6*H*,13*H*-5:12,7:14-di­methano­dibenzo[*d*,*i*][1,3,6,8]tetra­azecine

**DOI:** 10.1107/S1600536811033721

**Published:** 2011-08-27

**Authors:** Augusto Rivera, Mauricio Maldonado, Jaime Ríos-Motta, Karla Fejfarová, Michal Dušek

**Affiliations:** a Departamento de Química, Universidad Nacional de Colombia, Ciudad Universitaria, Bogotá, Colombia; b Institute of Physics ASCR, v.v.i., Na Slovance 2, 182 21 Praha 8, Czech Republic

## Abstract

The title compound, C_16_H_14_Cl_2_N_4_, is isomorphous with 2,9-dimethyl-6H,13*H*-5:12,7:14-dimethano­dibenzo[*d,i*]-[1,3,6,8]tetra­azecine [Rivera *et al.* (2009 ▶). *Acta Cryst.* E**65**, o2553] and has twofold symmetry, with two carbon atoms located on a twofold axis. Only van der Waals forces occur between molecules in the crystal. In the isomorphous compound the crystal structure is stabilized by weak C—H⋯π inter­actions.

## Related literature

For the isomorphous compound see: Rivera *et al.* (2009 ▶). For a related compound, see: Murray-Rust & Smith (1975 ▶). For uses of benzo-fused aminal cages, see: Schönherr *et al.* (2004 ▶); Polshettiwar & Varma (2008 ▶); Rivera *et al.* (2008 ▶).
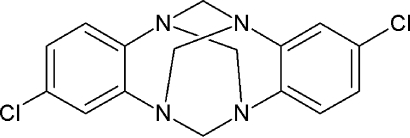


## Experimental

### 

#### Crystal data

C_16_H_14_Cl_2_N_4_
*M* *_r_* = 333.2
Orthorhombic, 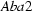

*a* = 9.8633 (6) Å
*b* = 19.0429 (14) Å
*c* = 7.6720 (7) Å
*V* = 1441.00 (19) Å^3^
*Z* = 4
Cu *K*α radiation
μ = 4.06 mm^−1^
*T* = 120 K
0.48 × 0.29 × 0.06 mm

#### Data collection

Agilent Xcalibur diffractometer with an Atlas (Gemini ultra Cu) detector
Absorption correction: analytical (*CrysAlis PRO*; Agilent Technologies, 2010 ▶)*T*_min_ = 0.291, *T*_max_ = 0.78
7379 measured reflections
1265 independent reflections
1174 reflections with *I* > 3σ(*I*)
*R*_int_ = 0.044

#### Refinement

*R*[*F* ^2^ > 2σ(*F* ^2^)] = 0.036
*wR*(*F* ^2^) = 0.095
*S* = 1.33
1265 reflections
101 parameters
H-atom parameters constrained
Δρ_max_ = 0.51 e Å^−3^
Δρ_min_ = −0.17 e Å^−3^
Absolute structure: (Flack, 1983 ▶), 569 Friedel pairs
Flack parameter: −0.03 (3)

### 

Data collection: *CrysAlis PRO* (Agilent Technologies, 2010 ▶); cell refinement: *CrysAlis PRO*; data reduction: *CrysAlis PRO*; program(s) used to solve structure: *SIR2002* (Burla *et al.*, 2003 ▶); program(s) used to refine structure: *JANA2006* (Petříček *et al.*, 2006 ▶); molecular graphics: *DIAMOND*(Brandenburg & Putz, 2005 ▶); software used to prepare material for publication: *JANA2006*.

## Supplementary Material

Crystal structure: contains datablock(s) global, I. DOI: 10.1107/S1600536811033721/bx2366sup1.cif

Structure factors: contains datablock(s) I. DOI: 10.1107/S1600536811033721/bx2366Isup2.hkl

Supplementary material file. DOI: 10.1107/S1600536811033721/bx2366Isup3.cml

Additional supplementary materials:  crystallographic information; 3D view; checkCIF report

## Figures and Tables

**Fig. 1.**
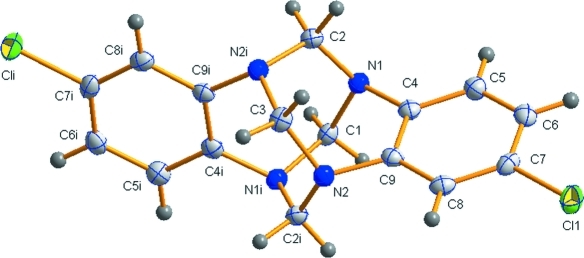
A view of the title compound with the numbering scheme. Displacement ellipsoids are drawn at the 50% probability level.

## supplementary crystallographic information

### Comment

Macrocyclic oligoaza compounds such as title compound (I) have been prepared in a variety of structural modifications and studied widely (Schönherr *et al.*, 2004). With regard to their use in the synthesis of ring-fused aminals which are of considerable interest as useful building block sand as potential drug candidates (Polshettiwar & Varma, 2008) we have used aromatic macrocyclic aminal compounds to perform one-pot synthesis of benzimidazole compounds (Rivera *et al.*, 2008). Engaged in the development of new synthetic pathways of ring-fused aminals, we undertaken the synthesis of the macrocyclic aminal 2,9-dichloro-6H,13*H*-5:12,7:14-dimethane- dibenzo[*d,i*][1,3,6,8]tetraazecine (I), by the reaction of 4-chloro-1,2-diaminobenzene with aqueous formaldehyde using a water-MeOH mixture as solvent. The title compound, shown in Fig. 1, is isomorphous with 2,9-dimethyl-6H,13*H*-5:12,7:14-dimethane- dibenzo[*d,i*][1,3,6,8]tetraazecine (Rivera *et al.*, 2009) and has twofold symmetry, with the C1 and C3 atoms located on a twofold axis and is The bond lengths and angles of the title compound are within normal ranges and are comparable with the isomorphous compound and with the related compound 6H,13*H*-5:12,7:14-dimethanedibenzo[*d,i*][1,3,6,8]tetraazecine (Murray-Rust & Smith, 1975) .However, the C6—C7 bond [1.369 (4) Å] in (I) is slightly shorter than that observed in the isomorphous structure [1.385 (3) Å Rivera *et al.*, 2009], suggesting some effect of halogen substitution. This fact is further supported by the C7—C8 bond length [1.403 (2) Å], which is slightly longer than C6—C7 bond [1.369 (4) Å].The crystal packing is stabilized by van der Waal's force. In the isomorphous compound the crystal structure is stabilized by weak C—H···π interactions.

### Experimental

A solution of 4-chloro-1,2-diaminobenzene (142 mg, 1 mmol) in MeOH/H_2_O (5 ml/15 mL) was added dropwise to an aqueous formaldehyde solution (5 ml, 37%) at 273 K. The mixture was allowed to stir for 1 h. at 273 K during which time a white solid was slowly deposited. After completion of the reaction title compound was obtained by filtration of the reaction mixture. The compound isolated was thoroughly washed with water and dried *in vacuo*. Slow evaporation of an ethyl acetate solution of the title compound yielded crystals suitable for single-crystal X-ray diffraction in 48% yield. Melting point 468 K.

### Refinement

All hydrogen atoms were placed in calculated positions with C–H distance 0.96 Å and refined as riding.The isotropic atomic displacement parameters of hydrogen atoms were evaluated as 1.2×*U*_eq_ of the parent atom.

### Crystal data

**Table:**

C_16_H_14_Cl_2_N_4_	*F*(000) = 688
*M**_r_* = 333.2	*D*_x_ = 1.536 Mg m^−^^3^
Orthorhombic, *A**b**a*2	Cu *K*α radiation, λ = 1.5418 Å
Hall symbol: A 2 -2ac	Cell parameters from 3667 reflections
*a* = 9.8633 (6) Å	θ = 4.5–66.8°
*b* = 19.0429 (14) Å	µ = 4.06 mm^−^^1^
*c* = 7.6720 (7) Å	*T* = 120 K
*V* = 1441.00 (19) Å^3^	Plate, colourless
*Z* = 4	0.48 × 0.29 × 0.06 mm

### Data collection

**Table:**

Agilent Xcalibur diffractometer with an Atlas (Gemini ultra Cu) detector	1265 independent reflections
Radiation source: Enhance Ultra (Cu) X-ray Source	1174 reflections with *I* > 3σ(*I*)
mirror	*R*_int_ = 0.044
Detector resolution: 10.3784 pixels mm^-1^	θ_max_ = 66.8°, θ_min_ = 6.5°
Rotation method data acquisition using ω scans	*h* = −11→11
Absorption correction: analytical (*CrysAlis PRO*; Agilent Technologies, 2010); analytical numeric absorption correction using a multifaceted crystal model	*k* = −22→22
*T*_min_ = 0.291, *T*_max_ = 0.78	*l* = −9→8
7379 measured reflections	

### Refinement

**Table:**

Refinement on *F*^2^	H-atom parameters constrained
*R*[*F*^2^ > 2σ(*F*^2^)] = 0.036	Weighting scheme based on measured s.u.'s *w* = 1/(σ^2^(*I*) + 0.0016*I*^2^)
*wR*(*F*^2^) = 0.095	(Δ/σ)_max_ = 0.004
*S* = 1.33	Δρ_max_ = 0.51 e Å^−^^3^
1265 reflections	Δρ_min_ = −0.17 e Å^−^^3^
101 parameters	Absolute structure: (Flack, 1983), 569 Friedel pairs
0 restraints	Flack parameter: −0.03 (3)
37 constraints	

### Special details

**Table:**

Refinement. The refinement was carried out against all reflections. The conventional *R*-factor is always based on *F*. The goodness of fit as well as the weighted *R*-factor are based on *F* and *F*^2^ for refinement carried out on *F* and *F*^2^, respectively. The threshold expression is used only for calculating *R*-factors *etc*. and it is not relevant to the choice of reflections for refinement.The program used for refinement, Jana2006, uses the weighting scheme based on the experimental expectations, see _refine_ls_weighting_details, that does not force *S* to be one. Therefore the values of *S* are usually larger than the ones from the *SHELX* program.

### Fractional atomic coordinates and isotropic or equivalent isotropic displacement parameters (Å^2^)

**Table:**

	*x*	*y*	*z*	*U*_iso_*/*U*_eq_	Occ. (<1)
Cl1	0.91895 (7)	0.18505 (3)	0.16603	0.0292 (2)	
N1	0.8773 (2)	0.47904 (11)	0.4178 (4)	0.0189 (6)	
N2	1.0725 (2)	0.44428 (10)	0.1595 (4)	0.0195 (6)	
C1	1	0.5	0.5138 (5)	0.0183 (11)	
C2	1.1711 (3)	0.46992 (13)	0.2877 (4)	0.0202 (7)	
C3	1	0.5	0.0621 (5)	0.0205 (11)	
C4	0.8852 (3)	0.40867 (13)	0.3509 (4)	0.0189 (7)	
C5	0.7937 (3)	0.35816 (14)	0.4088 (4)	0.0225 (7)	
C6	0.8032 (3)	0.28915 (15)	0.3488 (4)	0.0231 (8)	
C7	0.9055 (3)	0.27247 (14)	0.2356 (4)	0.0222 (8)	
C8	0.9965 (3)	0.32191 (13)	0.1713 (5)	0.0215 (7)	
C9	0.9845 (3)	0.39095 (14)	0.2278 (4)	0.0187 (7)	
H1a	1.022741	0.464287	0.59718	0.022*	0.5
H1b	0.977259	0.535713	0.59718	0.022*	0.5
H2a	1.212108	0.430722	0.346134	0.0243*	
H2b	1.248556	0.488821	0.228165	0.0243*	
H3a	1.060567	0.52114	−0.020563	0.0247*	0.5
H3b	0.939433	0.47886	−0.020563	0.0247*	0.5
H5	0.723842	0.370826	0.490066	0.027*	
H6	0.7394	0.254156	0.386118	0.0277*	
H8	1.065893	0.308653	0.089972	0.0258*	

### Atomic displacement parameters (Å^2^)

**Table:**

	*U*^11^	*U*^22^	*U*^33^	*U*^12^	*U*^13^	*U*^23^
Cl1	0.0363 (4)	0.0194 (3)	0.0318 (4)	−0.0013 (2)	0.0042 (3)	−0.0043 (3)
N1	0.0200 (11)	0.0177 (10)	0.0189 (11)	0.0005 (9)	0.0006 (10)	0.0007 (9)
N2	0.0201 (10)	0.0184 (10)	0.0199 (10)	−0.0004 (8)	0.0012 (10)	−0.0016 (11)
C1	0.0231 (19)	0.0156 (16)	0.0163 (19)	0.0021 (13)	0	0
C2	0.0174 (12)	0.0197 (12)	0.0236 (13)	0.0025 (10)	0.0004 (11)	−0.0009 (11)
C3	0.027 (2)	0.0215 (19)	0.014 (2)	0.0001 (14)	0	0
C4	0.0182 (13)	0.0213 (13)	0.0172 (12)	0.0006 (10)	−0.0040 (11)	0.0019 (11)
C5	0.0222 (14)	0.0245 (12)	0.0207 (13)	−0.0006 (10)	−0.0001 (13)	0.0012 (11)
C6	0.0217 (13)	0.0238 (13)	0.0237 (13)	−0.0031 (10)	−0.0054 (12)	0.0017 (12)
C7	0.0267 (15)	0.0169 (12)	0.0230 (13)	0.0021 (10)	−0.0073 (11)	−0.0016 (11)
C8	0.0214 (12)	0.0247 (12)	0.0186 (13)	0.0040 (9)	−0.0028 (15)	−0.0016 (12)
C9	0.0165 (12)	0.0216 (12)	0.0182 (12)	0.0004 (10)	−0.0026 (10)	0.0004 (10)

### Geometric parameters (Å, °)

**Table:**

N1—C1	1.472 (3)	C3—H3b	0.96
N1—C2^i^	1.473 (4)	C4—C5	1.392 (4)
N1—C4	1.437 (3)	C4—C9	1.402 (4)
N2—C2	1.467 (4)	C5—C6	1.395 (4)
N2—C3	1.482 (3)	C5—H5	0.96
N2—C9	1.435 (3)	C6—C7	1.369 (4)
C1—H1a	0.96	C6—H6	0.96
C1—H1b	0.96	C7—C8	1.391 (4)
C2—H2a	0.96	C8—C9	1.389 (4)
C2—H2b	0.96	C8—H8	0.96
C3—H3a	0.96		
			
C1—N1—C2^i^	115.28 (19)	N2—C3—H3a^i^	109.4707
C1—N1—C4	112.78 (19)	N2^i^—C3—H3a	109.4708
C2^i^—N1—C4	113.0 (2)	N2^i^—C3—H3b	109.4717
C2—N2—C3	114.81 (18)	H3a—C3—H3b	97.2479
C2—N2—C9	113.1 (3)	N1—C4—C5	119.7 (3)
C3—N2—C9	113.53 (18)	N1—C4—C9	120.2 (2)
N1—C1—N1^i^	119.9 (3)	C5—C4—C9	120.1 (2)
N1—C1—H1a	109.4716	C4—C5—C6	120.1 (3)
N1—C1—H1a^i^	109.4709	C4—C5—H5	119.9335
N1^i^—C1—H1a	109.4709	C6—C5—H5	119.9344
N1^i^—C1—H1b	109.4716	C5—C6—C7	118.5 (3)
H1a—C1—H1b	96.4816	C5—C6—H6	120.7546
N1^i^—C2—N2	117.3 (2)	C7—C6—H6	120.755
N1^i^—C2—H2a	109.4713	C6—C7—C8	122.9 (3)
N1^i^—C2—H2b	109.4717	C7—C8—C9	118.4 (3)
N2—C2—H2a	109.471	C7—C8—H8	120.82
N2—C2—H2b	109.4709	C9—C8—H8	120.8182
H2a—C2—H2b	100.2895	N2—C9—C4	119.9 (2)
N2—C3—N2^i^	119.4 (3)	N2—C9—C8	120.3 (3)
N2—C3—H3a	109.4717	C4—C9—C8	119.8 (3)
			
?—?—?—?	?		

Symmetry codes: (i) −*x*+2, −*y*+1, *z*.

### Hydrogen-bond geometry (Å, °)

**Table:**

*D*—H···*A*	*D*—H	H···*A*	*D*···*A*	*D*—H···*A*
?—?···?	?	?	?	?
